# C_60_ ions of 1 MeV are *slow* but elongate nanoparticles like *swift* heavy ions of hundreds MeV

**DOI:** 10.1038/s41598-019-49645-5

**Published:** 2019-10-18

**Authors:** H. Amekura, K. Narumi, A. Chiba, Y. Hirano, K. Yamada, D. Tsuya, S. Yamamoto, N. Okubo, N. Ishikawa, Y. Saitoh

**Affiliations:** 1 National Institute for Materials Science (NIMS), Tsukuba, Ibaraki Japan; 2 National Institutes for Quantum and Radiological Science and Technology (QST), Takasaki, Japan; 3 Japan Atomic Energy Agency (JAEA), Tokai, Ibaraki Japan

**Keywords:** Nanoparticles, Atomic and molecular collision processes

## Abstract

This study reports that high fluence fullerene ion (C_60_^+^) irradiation of 1–6 MeV, which was made possible by a new-type of high-flux ion source, elongates metal nanoparticles (NPs) in amorphous SiO_2_ as efficiently as swift heavy ions (SHIs) of 200 MeV Xe^14+^, i.e., two orders of the magnitude higher energy ions. Comparing the irradiation effects induced by both the beams, the stopping processes of C_60_ ions in SiO_2_ are discussed in this paper. Despite of having almost the same elongation efficiency, the C_60_^+^ irradiation induced ~10 times more efficient sputtering due to the clustering enhancement and/or the synergy effect. Ion tracks of ~10.4 nm in diameter and 60–80 nm in length were observed in crystalline SiO_2_ under 4 MeV C_60_ irradiation_._ While the track diameter was comparable to those by SHIs of the same electronic stopping, much shorter track lengths than those predicted by a rigid C_60_ molecule model indicates that the fragmentation occurred due to nuclear collisions. The elongation of the metal NPs was induced only down to the depth where the tracks were observed but not beyond.

## Introduction

Modification of materials using swift heavy ions (SHIs), i.e., high energy ions with stopping power in materials that are primarily dominated by electronic forces, has recently received an increasing amount of attention^1^. The examples include the anisotropic deformation of amorphous metals/glasses^2,3^, shape elongation of metal nanoparticles (NPs) embedded in materials^4–15^, and etched ion-track engineering^16^. When NPs embedded in SiO_2_ are irradiated with SHIs, the NPs are elongated toward the direction parallel to the SHI beam. Many impacts of SHIs gradually transform originally-spherical NPs to nano-rods. This paper describes that the same deformation is also induced by 1–6 MeV fullerene (C_60_) ions, while such C_60_ ions are not classified in SHIs because of their slow velocities. The energy per nucleon of several MeV C_60_ ion, which is an index of ion velocity, is on the order of 10^−3^ MeV/u, while that of SHI is ~1 MeV/u or higher. However, C_60_ ions provide high electronic energy deposition comparable to SHIs due to the coincident impacts of 60 carbon atoms within a limited area of the C_60_ molecule size of ~0.7 nm in diameter. The stopping powers of the C_60_ ion is approximated as the sum of sixty independent carbon monomer ions with the energy of each one (*E*/60) expressed as,

1 $${S}_{i}(E,\,{{\rm{C}}}_{60})=60\,{S}_{i}(E/60,\,{{\rm{C}}}_{1})$$

where *i* = *n* (nuclear) or *e* (electronic)^17^. The monomer stopping power *S*_i_ (*E*/60, C_1_) is derived from SRIM 2013 code^18^. A 6 MeV C_60_ ion provides ~15.5 keV/nm of electronic stopping power *S*_e_ in amorphous SiO_2_, which is as high as *S*_e_ of 200 MeV Xe ion (15.0 keV/nm), as shown in Table 1. In this paper, C_60_ ions of 1–6 MeV were applied, which correspond to *S*_e_ of 6.3–15.5 keV/nm. Using the same *S*_e_ range of SHIs, we have already succeeded in the elongation of NPs as shown in Table 1^4^.

**Table 1:** Electronic and nuclear stopping powers (*S*_e_, *S*_n_) at the surface of amorphous SiO_2_ calculated from SRIM2013 code for SHIs and from the Eq. (1) for cluster ions. The elongation efficiency is defined as the inverse of the fluence where the optical dichroism becomes 0.02.

Ion energy *E*	Ion species	*S*_e_ (keV/nm)	*S*_n_ (keV/nm)	*E*/*M* (MeV/u)	Elongation efficiency (×10^−14^ cm^2^)	Ref.
200 MeV	Xe^14+^	15.0	0.051	1.50	175	^4^
6 MeV	C_60_^+^	15.5	2.66	8.33 × 10^−3^	163	this work
4 MeV	C_60_^+^	12.7	3.41	5.56 × 10^−3^	226	this work
2 MeV	C_60_^+^	9.3	4.83	2.78 × 10^−3^	85.5	this work
1 MeV	C_60_^+^	6.3	6.18	1.39 × 10^−3^	52.1	this work
200 MeV	Au^13+^	17.7	0.142	1.02	144	^4^
60 MeV	Ti^5+^	5.9	0.011	1.25	46.7	^4^
50 MeV	Si^4+^	3.2	0.004	1.78	16.7	^4^
8 MeV	Si^3+^	3.2	0.017	0.285	28.5	^4^
140 MeV	Si^12+^	2.2	0.001	4.98	2.55	^4^
1.7 MeV	Si^+^	1.6	0.055	0.0605	1.49	^4^
100 keV (6 MeV/60)	C^+^	0.258	0.0443	8.33 × 10^−3^	—	—
66.7 keV (4 MeV/60)	C^+^	0.212	0.0568	5.56 × 10^−3^	—	—

Since 1990s, C_60_ ions of up to 40 MeV have been available at the Orsay facility, France^19–21^. However, the available fluences have been limited because of the extremely low fluxes on the order of 10^6^ C_60_/cm^2^s^21^. Conversely, obvious NP elongation was thought to require high fluences as 10^13^ C_60_^+^/cm^2^ or more, i.e., irradiation time of ~4 months. The elongation experiments have only recently become possible because the Takasaki group, a part of the research team that conducted this study, has developed an electron-attachment-type C_60_ ion source which generates enormous fluxes of ~10^10^ C_60_/cm^2^s and consequently attains the high fluences^22^.

Using the high-flux C_60_ ion source, we have firstly succeeded in elongating the shape of NPs by the cluster ion irradiation. Simultaneously we observed different phenomena which have not been observed under SHI irradiation, i.e., the formation of short ion tracks, and significantly enhanced sputtering, and unexpectedly weak velocity effect. In this paper, the interplay between these phenomena and NP elongation are also discussed.

## Results

### Elongation of NPs: TEM observation

**Figure 1:**
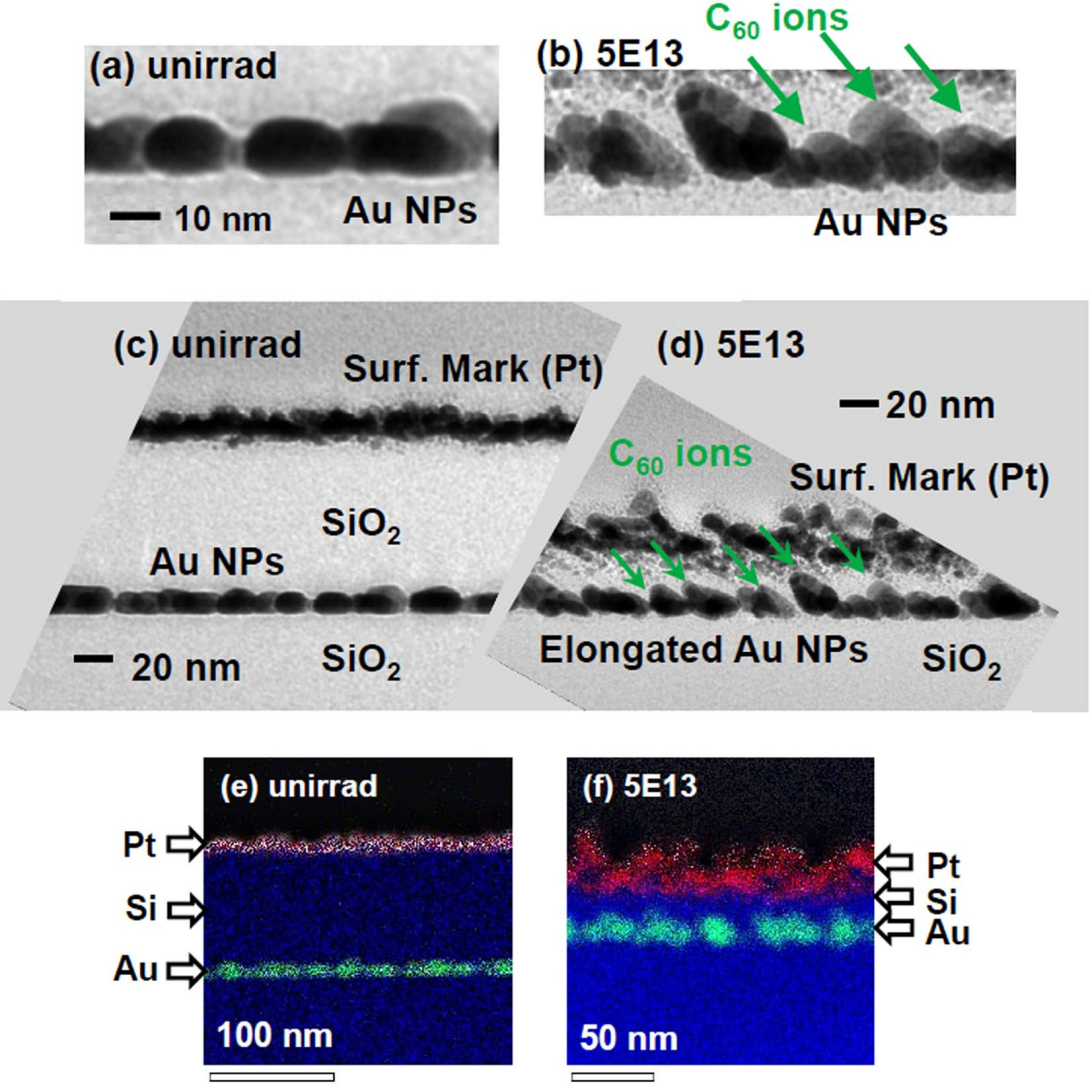
Cross-sectional TEM images of Au NPs embedded in SiO_2_ before (**a**,**c**,**e**) and after (**b**,**d**,**f**) irradiation with 4 MeV C_60_ ions at a fluence of 5 × 10^13^ C_60_^+^/cm^2^. Enlarged images of Au NPs (**a**,**b**) and overviews of deposited SiO_2_ layer on the NPs (**c**,**d**). A thin layer of Pt was deposited for surface marker. (**a**–**d**) Bright field (BF) images and (**e**,**f**) STEM-EDS element mappings. In (**e**,**f**), red, blue, and green regions correspond to Pt, Si, and Au rich regions.

Figures 1(a–d) show bright field (BF) cross-sectional TEM (XTEM) images of Au NPs embedded in SiO_2_, (a)(c) before and (b)(d) after the irradiation with 4 MeV C_60_^+^ ions to a fluence of 5 × 10^13^ C_60_^+^/cm^2^. The incident angle of the C_60_^+^ beam was set to 45°, as shown by the arrows in Figs. 1(b,d). Before the sample thinning, a thin Pt layer was deposited as a surface marker. In unirradiated state (Figs. 1(a,c)), a monolayer of Au NPs was observed, each of which exhibited an *oblate* spheroidal shape with principle axis pointing to *the surface normal* (see supplementary materials). The Pt marker was observed on the top of a *100 nm* thick SiO_2_ layer over the Au NP layer as shown in Fig. 1(c). After the irradiation with 5 × 10^13^ C_60_^+^/cm^2^ (Fig. 1(d)), the thickness of the deposited SiO_2_ layer markedly decreased from ~100 nm to *~30 nm* due to enhanced sputtering associated with the C_60_ irradiation. Consequently, the distance between the Pt marker and Au NPs considerably decreased, which could make it difficult to distinguish between the markers and NPs, though scanning TEM and X-ray energy-dispersive spectrometry (STEM-EDS) mapping made it possible to clearly distinguish the Pt marker and Au NPs, as shown in Figs. 1(e,f).

The widely distributed markers in Fig. 1(d) manifested a very rough surface caused by the sputtering. Figures 1(b,d) clearly shows that the Au NPs raised the major axes toward a 45° angle, which was the incidence angle of the ion beam. In addition, the NPs changed the shapes to nearly *prolate* spheroids with the principle axes pointing in the direction of the 45° beam angle. These observations have clearly indicated that the shape elongation of NPs was induced by the slow and low energy 4 MeV C_60_^+^ ions.

### Elongation of NPs: optical dichroism

**Figure 2:**
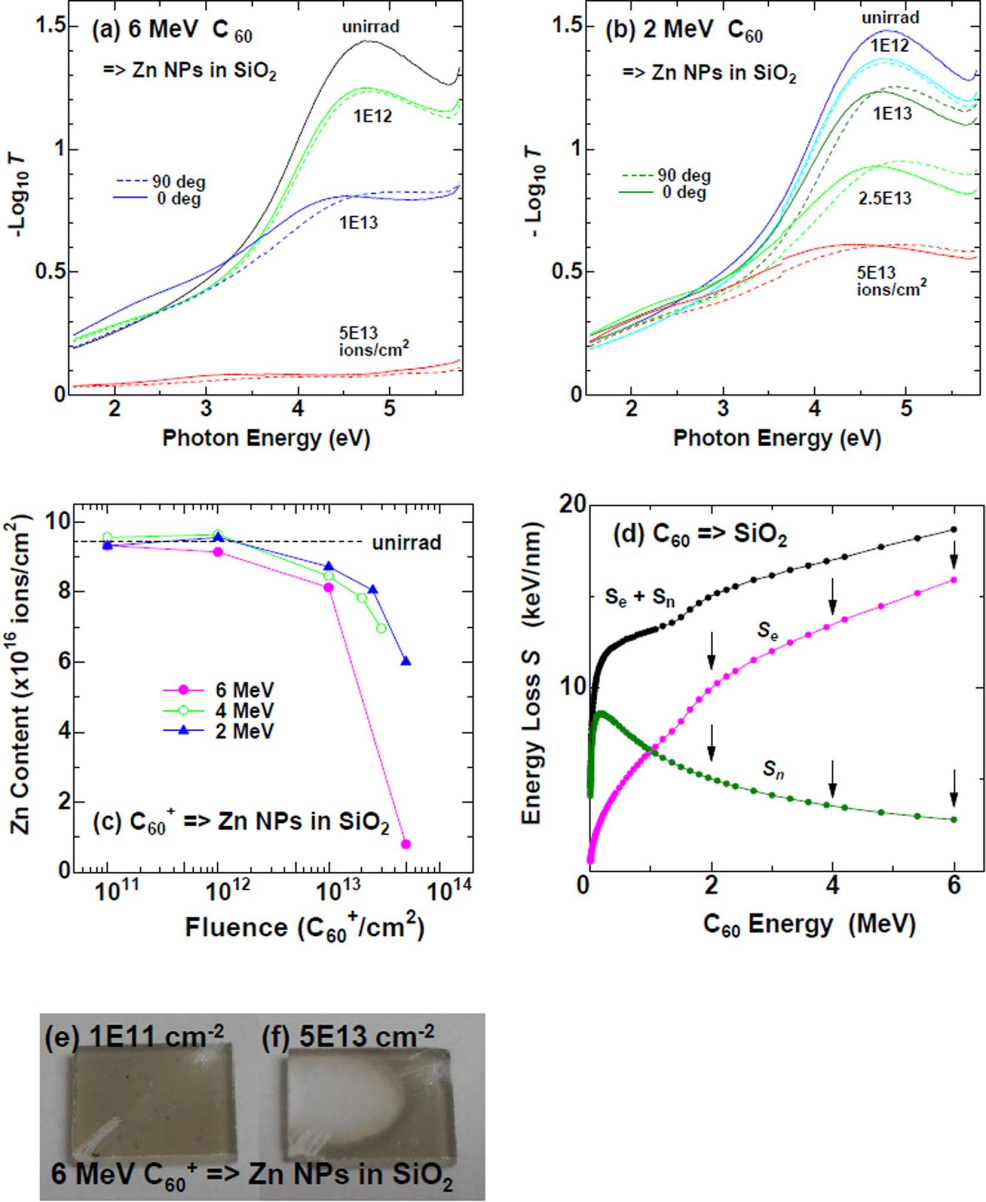
(**a**,**b**) Optical absorption spectra of embedded Zn NP samples detected by linearly polarized light, which were irradiated with 6 MeV and 2 MeV C_60_^+^ ions at various fluences indicated in the figures. The transmittance of light is denoted by *T*. The light polarization angle was 0° (solid curves) when the polarization plane included the major axes of the NPs. (**c**) Fluence dependence of Zn content in the samples irradiated with C_60_^+^ ions of 2 MeV (triangles), 4 MeV (open circles), and 6 MeV (closed circles), detected by RBS. (**d**) Energy dependence of nuclear stopping power (*S*_n_) and electronic stopping power (*S*_e_) of C_60_ ions in SiO_2_, calculated by SRIM 2013 code using Eq. (1). (**e**,**f**) Photoimages of Zn NP samples irradiated with 6 MeV C_60_^+^ ions at a fluence of 1 × 10^11^ C_60_^+^/cm^2^ and 5 × 10^13^ C_60_^+^/cm^2^, respectively. The dark brown color is due to Zn NPs dispersed in SiO_2_. A colorless region in the sample (**f**) indicated that Zn NPs were mostly lost due to sputtering by C_60_ irradiation, which was also confirmed by RBS.

Optical absorption spectra of Zn NP samples^23,24^ irradiated with C_60_ ions to various fluences were detected under linearly polarized light illumination. The polarization angle is 0° when the polarization plane includes the major axes of the elongated NPs. As reported in ref.^4,11^, the difference in the optical absorption at 0° polarization and 90° polarization is, in certain conditions, proportional to the aspect ratio (AR) of the elongated NPs. The absorption spectra at 0° (solid curves) and 90° (broken curves) polarization at various fluences are presented in Fig. 2(a,b) for 6 MeV (*S*_e_ = 15.5 keV/nm) and 2 MeV (9.3 keV/nm) C_60_ ions, respectively. At both the ion energies, the *S*_e_ was high enough to induce NP elongation if it was supplied from SHIs as shown in Table 1.

The dependence between 2 and 6 MeV ions is qualitatively similar but not quantitatively. In an unirradiated state, both spectra detected at 0° and 90° polarization fall on the same curve. However, as the fluence increased, spectral deviation between 0° and 90° polarization, i.e., the difference between the solid and broken curves increased up to 1 × 10^13^ C_60_^+^/cm^2^. Simultaneously, the absorption peak intensity at ~4.8 eV decreased with increased fluence, which can be attributed to sputtering loss of Zn atoms from the samples. The absorption mostly disappeared at 5 × 10^13^ C_60_^+^/cm^2^ for 6 MeV but a half of the peak remained for 2 MeV. According to Rutherford backscattering spectrometry (RBS) measurements combined with Rump code analysis^25^, more than 90% and 40% of Zn atoms were lost from the samples at the fluence of 5 × 10^13^ C_60_/cm^2^ with 6 MeV and 2 MeV C_60_ ions, respectively (Fig. 2(c)). Figures 2(e,f) show images of the samples irradiated with 6 MeV ions at 1 × 10^11^ and 5 × 10^13^ C_60_^+^/cm^2^. While Zn NPs in SiO_2_ show brown color (Fig. 2(e)), a colorless region was clearly observed at 5 × 10^13^ C_60_^+^/cm^2^, as shown in Fig. 2(f), indicating the severe loss of Zn NPs caused by sputtering. Samples irradiated with 2 MeV C_60_ ions displayed lower efficiencies with respect to both the NP elongation and the sputtering loss.

**Figure 3:**
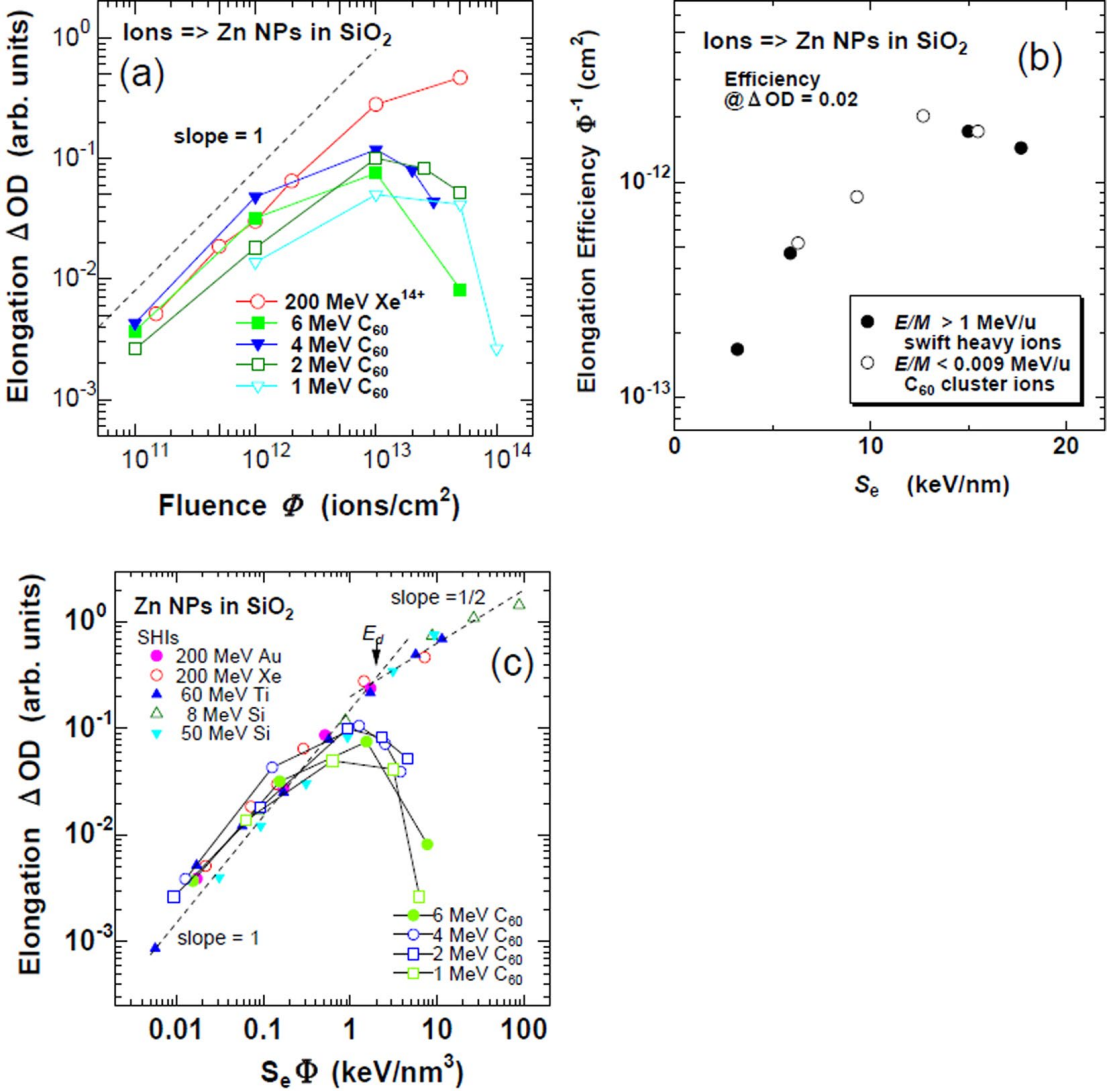
Elongation of Zn NPs embedded in SiO_2_ was determined by optical dichroism spectroscopy. (**a**) Fluence dependences of the elongation induced by Xe ions of 200 MeV, and C_60_ ions of 6 MeV, 4 MeV, 2 MeV, and 1 MeV, respectively. A broken line indicates the slope of unity. (**b**) *S*_e_ dependence of the elongation efficiencies of Zn NPs for C_60_ ions of 1, 2, 4, and 6 MeV, i.e., *E/M* < 0.009 MeV/u (open circles) and for swift heavy ions (>1 MeV/u), i.e., 200 MeV Au^13+^, 200 MeV Xe^14+^, 60 MeV Ti^6+^, and 50 MeV Si^5+^ (closed circles). (**c**) The elongation is plotted against the product of the electronic stopping power and the fluences, i.e., the electronic deposited energy density.

The fluence dependence of NP elongation was determined from optical spectra using the method described in^4^ and is presented in Fig. 3(a), with the SHI data of 200 MeV Xe ions for reference. In the low fluence region between 1 × 10^11^ and 1 × 10^12^ C_60_^+^/cm^2^, 4 MeV (*S*_e_ = 12.7 keV/nm) and 6 MeV (15.5 keV/nm) C_60_ irradiation induced shape elongation comparable to or slightly higher than 200 MeV Xe irradiation (15.0 keV/nm), while irradiation at 1 and 2 MeV induced less elongation. However, it should be noted that *the elongation is induced with even 1 MeV C*_60_ *ions* (*S*_e_ = 6.3 keV/nm). It is not surprising because the 1^st^ threshold of the shape elongation by SHIs is ~3 keV/nm^4^. The 1 MeV C_60_ ions have higher *S*_e_ than the threshold. It is noted that the elongation is induced by SHIs with much lower efficiency even below the 1^st^ threshold^4^. The slightly higher efficiency of 4 and 6 MeV C_60_ ions compared to 200 MeV Xe ions could be attributed to two: (i) The much slower velocity of the cluster ions compared to SHIs, which reduces the energy of δ-rays and excitation volume, resulting in a higher excitation density^26^. (ii) The synergy effect between *S*_n_ and *S*_e_. While *S*_n_ is much lower than *S*_e_ for SHIs, this is not the case for MeV C_60_ ions, as shown in Table 1. Consequently the synergy effect is not excluded.

From the slope of unity in the log-log plot in Fig. 3(a), the NP elongation linearly increased with the fluence up to 1 × 10^12^ C_60_^+^/cm^2^. However, the slope was reduced to less than unity above 1 × 10^12^ C_60_^+^/cm^2^, and finally turned to a negative slope around 1 × 10^13^ C_60_^+^/cm^2^. The sublinear increase in intermediate fluences and the decrease in high fluences can be attributed to the destruction of the elongated NPs caused by the enhanced sputtering of the cluster ions. In fact, the quantities of Zn atoms in the samples decreased with the fluence as evidenced by RBS, as shown in Fig. 2(c).

To compare the elongation efficiency between the different beams, the efficiency was defined as the inverse of the fluence, where the optical elongation reaches to the value of 0.02, and shown in Table 1 and Fig. 3(b). See details for ref.^4^. (While the fluence at which the optical elongation reached to 0.10 was used in ref.^4^, the fluence at which the elongation reached to 0.02 was used in this article, because the linear region was limited.)

As shown in Fig. 3(b), the maximum efficiency was obtained under 4 MeV C_60_ ion irradiation. However, both the data by SHIs (closed circles) and by C_60_ ions (open circles) fall on almost the same dependence, indicating that large difference due to the velocity effect was not observed.

In the previous literature, the elongation induced by SHI irradiations is normalized when the abscissa is plotted with the product of *S*_e_ and the fluence Φ. Therefore the elongation is determined only by the electroni deposited energy density^4^. Of course, this kind of simple relation is not applied to sputtering induced by SHIs^27^. To check whether this simple relation is hold in this case or not, the optically detected elongation was plotted with the product of *S*_e_ and the fluence Φ. In contrast to SHIs, the data points do not fall on the same curve. A simple relationship like the elongation induced by SHI irradiation was not observed, probably this process included the strong electronic sputtering.

### Ion track formation

**Figure 4:**
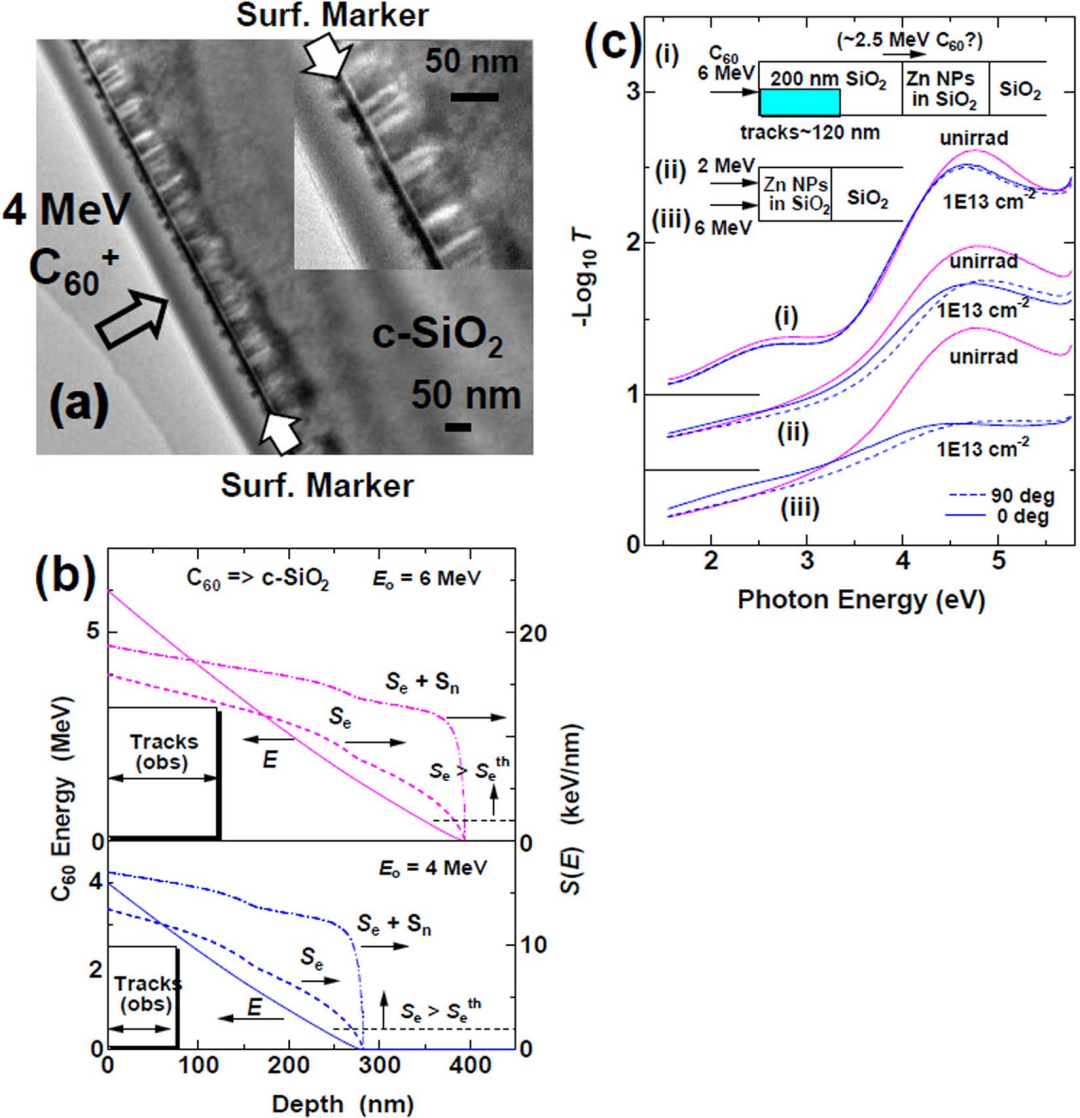
(**a**) A bright field cross-sectional TEM image of c-SiO_2_ irradiated with 4 MeV C_60_^+^ ions at a fluence of 5 × 10^11^ C_60_^+^/cm^2^ with an incident angle of 7° from the surface normal. A thin layer of Pt was deposited as a surface marker after irradiation. The inset shows an expanded image. (**b**) Depth dependence of C_60_ ion energy injected into crystalline SiO_2_ with incident energy of 4 MeV (lower) and 6 MeV (upper), calculated from Eq. (2). Depth dependences of electronic stopping power *S*_e_ and of total stopping power, *S*_e_ + *S*_n_, are plotted by broken lines and chain lines, respectively. Experimentally observed track lengths, and the threshold *S*_e_ for track formation in c-SiO_2_ of *S*_e_^th^ = 2 keV/nm^30^, are also shown in the figures. (**c**) The effects of a deposited SiO_2_ layer of 200 nm thick on a Zn NP sample are monitored by linearly polarized absorption spectra. The spectra (i) were detected from the Zn NP sample with the deposited layer, which were irradiated with 6 MeV C_60_ ions to 1 × 10^13^ C_60_^+^/cm^2^. In this configuration, the ion tracks do not reach the Zn NP layer, while calculations estimate that the Zn NPs could be irradiated with ~2.5 MeV C_60_ ions through the deposited layer if the C_60_ ions would not be fragmented. The spectra (ii) and (iii) were detected from the Zn NP samples without the deposited layer, irradiated with 2 MeV and 6 MeV C_60_ ions, respectively. Polarization angle of 0° (solid curves) is defined when the polarization plane includes major axes of NPs, while that of 90° (broken curves) is defined when the plane is perpendicular to the axes.

Although the mechanism of NP shape elongation induced by SHIs is yet to be identified, a majority of researchers agree with the importance of the ion-track formation in matrix materials^4–15^. However, the tracks cannot be directly observed by TEM in amorphous matrices^28^, except in a very thin self-standing film^29^. For reference, a piece of crystalline SiO_2_ (c-SiO_2_) without NPs was irradiated with the C_60_ ions and observed by XTEM. Figure 4(a) presents an XTEM image of the sample irradiated with 4 MeV C_60_ ions at a fluence of 5 × 10^11^ C_60_^+^/cm^2^. The incident angle of the C_60_ beam was set to 7° from the surface normal to avoid channeling. The irradiated surface was coated by a thin Pt layer as a surface marker for XTEM analysis. It should be noted that the surface was much flatter than that in Fig. 1(b) because the fluence was much lower.

Many cylindrical structures of 10.4 ± 3.0 nm in diameter and 60–80 nm in length were observed below the black surface marker layer in Fig. 4(a). The observed track length of 60–80 nm was unexpectedly short. It was experimentally observed that the track length increased when the ions were irradiated exactly at the surface normal, i.e., 0°, probably due to channeling. The energy of the C_60_ ion at depth *x* in SiO_2_ was calculated based on the rigid C_60_ molecule model, i.e., the fragmentation of C_60_ molecule is excluded,

2 $$E(x,{{\rm{C}}}_{60})={E}_{o}-{\int }_{0}^{x}[{S}_{e}(E,{{\rm{C}}}_{60})+{S}_{n}(E,{{\rm{C}}}_{60})]dx,$$

where *E*_o_ is the incident energy of the C_60_ ion. The results for 4 and 6 MeV ions are shown in Fig. 4(b). The stopping powers *S*_e_(*E*) + *S*_n_(*E*) and *S*_e_(*E*) are provided in Fig. 4(b). Since the threshold electronic stopping power for track formation in c-SiO_2_ is known to be ~2 keV/nm^30^, tracks by 4 MeV C_60_ ions would be formed down to a depth of 270 nm, where *S*_e_ > 2 keV/nm. It should be noted again that the experimentally observed track lengths were 60–80 nm, much shorter than the rigid model predicted.

As more clearly shown in the inset of Fig. 4(a), dot-like structures are observed on the vacuum side of the surface marker layer. Judging from the intensity of the dot images, they are likely from Pt, but were probably pushed toward the vacuum side by the hillocks of c-SiO_2_ underneath. The hillocks are indirectly observed in this case. In fact, it is clearly observed that the lateral dimensions of the dots are larger the diameters of the tracks inside of c-SiO_2_, because of the thickness of the deposited Pt marker. The hillocks are most likely formed when the tracks met the surface^31^.

### Shape elongation deeper than the track lengths

The next question is whether NP elongation was only induced within the track depth or also beyond. A 200 nm thick layer of amorphous SiO_2_ was deposited on a Zn NP sample, irradiated with 6 MeV C_60_ ions, and compared with samples without the deposited layer. As shown in Fig. 4(c), a weak hump was observed at ~2.6 eV for a sample with the deposited layer (curves (i)), which was attributed to an interference fringe in the deposited layer.

The 200 nm thickness was wider than the typical track length of ~120 nm for 6 MeV C_60_ ions. In this configuration, Zn NPs did not interact with the ion tracks unless the deposited layer was severely sputtered. According to Fig. 4(b), the mean energy of C_60_ ions was reduced from 6 MeV to ~2.5 MeV after passing through the 200 nm thick SiO_2_ layer. The energy of ~2.5 MeV was still high enough to induce NP elongation since the anisotropic absorption, i.e., elongation was observed even under 2 MeV C_60_ irradiation, as shown by curve (ii) in Fig. 4(c) and also Fig. 2(b). It should be noted that the direct irradiation of the Zn layer with 2 MeV C_60_ ions induced higher elongation than indirect irradiation with ~2.5 MeV C_60_ ions through the 200 nm-thick deposited layer, although the latter had higher energy. This observation can be interpreted that the 6 MeV C_60_ ion lost the energy going through the deposited layer of 200 nm thick and was fragmented. The fragmented carbon ions which totally have ~2.5 MeV no longer to form ion tracks nor elongation of NPs. Probably the distances between the fragments become too long to induce the cooperative effect between them. However, since the track length of ~120 nm was determined from quartz but not from amorphous SiO_2_, further confirmation is necessary.

## Discussion

### Enhanced sputtering

It is well known that there are two types of sputtering mechanisms: nuclear and electronic sputtering. Furthermore, the synergy effect between both types could be possible^32^. Since the present case is in an intermediate energy region, we evaluated which mechanism, i.e., *S*_n_ or *S*_e_ was dominant for the observed enhanced sputtering under C_60_ irradiation.

Energy dependences of stopping powers, *S*_e_ and *S*_n,_ of C_60_ ions are plotted in Fig. 2(d), indicating that *S*_e_ was higher than *S*_n_ when *E* > 1 MeV. However, *S*_n_ was *not* negligible, which is different from SHIs. An important observation was revealed in this energy region, namely *S*_*e*_ *increased but S*_*n*_ *decreased with ion energy*. Figure 2(c) shows the fluence dependence of the Zn loss induced by C_60_ ion irradiation at three different energies, 2, 4, and 6 MeV. The Zn loss was more efficient at higher energies. The energy dependence of the sputtering efficiency and that of *S*_e_ and *S*_n_ are presented in Table 2. The electronic stopping power *S*_e_ and the observed sputtering yield increased with increasing the energy, while the nuclear stopping power *S*_n_ decreased. This difference in energy dependence indicated that sputtering can be attributed to an *S*_e_-related mechanism, not an *S*_n_-related one.

**Table 2:** Energy dependence of sputtering efficiency (observation) and of electronic and nuclear stopping powers calculated.

With increasing the ion energy *E*		Ref.
electronic stopping *S*_e_	increases	Fig. 2(d)
nuclear stopping *S*_n_	decreases	Fig. 2(d)
observed sputtering	increases	Fig. 2(f)
=> positive correlation between the observed sputtering and *S*_e_.		

A further quantitative evaluation is presented in Table 3. The sputtering yield of SiO_2_ from C_60_ ion irradiation was estimated using both the models. In the nuclear sputtering model, the nuclear sputtering yield *Y*_n_ by a C_60_ ion with energy *E* is given by a sum of those independent sixty C monomer ions with *E*/60,

3 $${Y}_{n}(E,{{\rm{C}}}_{60})=60{Y}_{n}(E/60,\,{{\rm{C}}}_{1})$$

The sputtering yield of SiO_2_ by the C monomer ion, *Y*_n_ (*E*/60, C_1_), was determined by SRIM 2013 code. The yield was 16.3 atoms/C_60_-ion for 6 MeV C_60_ ions.

**Table 3:** Comparison of the experimental sputtering yield with the nuclear and the electronic sputtering models.

	Model	4 MeV	6 MeV
Nuclear sputtering model	60 times of the sputtering yield of C-monomer-ions calculated from SRIM code. (atoms/C_60_ ion)	22.3	16.3
Electronic sputtering model	With presuming a C_60_ ion as a swift heavy monomer ion, an experimental sputtering law for swift heavy ion [*] was applied. (atoms/C_60_ ion)	410	745
Experiment	Sputtered thickness of the film at the fluence of 5 × 10^13^ C_60_/cm^2^.	~70 nm (Fig. 1(d))	>70 nm (Fig. 2(e))
	Experimental Sputtering Yield (atoms/C_60_)	~3.2 × 10^3^	>3.2 × 10^3^

*N. Matsunami *et al*. Nucl. Instr. Meth. Phys. Res. B209, 288 (2003).

In the electronic sputtering model, a C_60_ ion, which is characterized as *S*_e_, is treated as a swift heavy ion. Applying an experimental cubic formula for SiO_2_,

4 $${Y}_{e}={B}_{1}{S}_{e}^{3},$$

where *B*_1_ = 0.20 atoms/ion (nm/keV)^3^ ^27^, the yield of 745 atoms/C_60_-ion was estimated for 6 MeV C_60_ ion. As shown in Fig. 1, the thickness of the SiO_2_ top-layer decreased from 100 to 30 nm under 4 MeV C_60_ irradiation at a fluence of 5 × 10^13^ C_60_^+^/cm^2^. Also as shown in Fig. 2(f), the 70 nm of thick Zn NP layer was completely sputtered out under 6 MeV C_60_ irradiation, below 5 × 10^13^ C_60_^+^/cm^2^. The observed sputtering yields were 3.2 × 10^3^ and >3.2 × 10^3^ atoms/C_60_ for 4 MeV and 6 MeV irradiation, respectively, both of which were much higher than the calculated values. The sputtering yield was greatly enhanced probably due to cluster enhancement or the synergy effect^17,33,34^.

### Track diameter

As shown in Fig. 4(a), the track diameter of 10.4 ± 3.0 nm was determined in crystalline SiO_2_ under 4 MeV C_60_^+^ irradiation (*S*_e_ = 12.7 keV/nm). Afra *et al*. summarized various experimental data of *S*_e_ dependence of the track sizes of crystalline SiO_2_ with the i-TS calculations of the velocities of 0.5 MeV/u and 7.0 MeV/u^30^. The calculated track diameters for 15.3 keV/nm (4 MeV C_60_^+^ in c-SiO_2_) were ~11 nm and ~9 nm for the low and high velocities, respectively. The track radius of the 4 MeV C_60_^+^ ion in c-SiO_2_ was not so large compared to SHIs. Kluth *et al*. evaluated *amorphous* SiO_2_ by small angle X-ray scattering (SAXS) and reported the total track diameters of ~10 nm for low velocity and ~8 nm for high velocity, respectively^28^. It should be noted that tracks in amorphous SiO_2_ have core/shell structures^15^, which is different from the hard-cylinder-type of crystalline SiO_2_^30^.

P. Kumar *et al*. have studied the *S*_e_ dependence of track diameter in C_60_ films using TEM observation under various ions (*S*_e_ = 0.04–40.8 keV/nm) irradiations. They observed the largest ion track of ~20 nm in diameter under 30 MeV C_60_ irradiation. Furthermore, they observed that the diameter increased in proportional to *S*_e_, which was different from the conventional one that the square of the diameter is proportional to the *S*_e._ To explain the dependence, Kumar *et al*., proposed the Coulomb explosion model for the track formation in C_60_ films^35^. Kitayama *et al*. studied the core/shell ion tracks in amorphous SiN film by C_60_ ion irradiation. The total and core diameters did not depend on *S*_e_ and were almost constant of 11.2 nm and 4.3 nm between *S*_e_ = 3 and 22 keV/nm^36^. Relatively large but unusual energy dependent track formations were reported, which could be related to the synergy effect between *S*_e_ and *S*_n_.

### Possible applications

Another concern, i.e., rather pragmatic motivation, of this study is possible substitutability of swift heavy ions (SHIs) by MeV C_60_ ions. Acceleration of SHIs requires the world-class big accelerator facilities. The number of the facilities available for materials science applications is quite limited, e.g., a few facilities in Japan. Since a few to several MeV C_60_ ions provide high electronic energy deposition comparable to SHIs, they could be used as substitution of SHIs. The acceleration energy of a few MeV is not practically high. It is easily attainable in quite common accelerator facilities with the terminal voltage of ~1–2 MV, which are found at many places. If the newly-developed high flux C_60_ ion sources can be combined with the commonly-used ~1 MV accelerator facilities, the high-density electronic excitation which are comparable to SHIs would be easily accessible in quite many facilities, i.e., more than 20 facilities in Japan.

However, some irradiation effects of MeV C_60_ ions are different from those of SHIs. Ion ranges of SHIs are e.g., ~10 μm, much longer than those of MeV C_60_ ions, even assuming the ion range of C_60_ ion as that of constitute C monomer ion, e.g., ~a few hundred nm. As shown in this paper, the depths where the high *S*_e_ is available are further limited, by the fragmentation of C_60_ ions. The cluster enhanced sputtering cannot be neglected. The perfect substitution of SHIs by MeV C_60_ ions is difficult. However, substitution of certain aspects could be possible. To understand the substitutability, we are studying the interaction between MeV C_60_ ions and materials, in this case, NPs.

## Summary

Fullerene ions of 1–6 MeV induce dense electronic excitation in SiO_2_ comparable to swift heavy ions of 200 MeV Xe^14+^. Shape elongation of Au and Zn NPs was confirmed in amorphous SiO_2_ under the C_60_^+^ ion irradiation. According to the optical dichroism spectroscopy, 4 MeV and 6 MeV C_60_ ion irradiation induced Zn NP shape elongation with a high efficiency at low fluences, which was comparable to or slightly higher than 200 MeV Xe ions. While the *S*_e_ of 6 MeV C_60_ ions and that of 200 MeV Xe ions were virtually the same, the slower velocity of the former reduced the δ-ray energy, which resulted in the enhancement of the excitation density^26^. While the NP elongation linearly increased with the fluence up to 1 × 10^13^ Xe^14+^/cm^2^ for 200 MeV Xe ions, the elongation by C_60_ ions sublinearly increased exceeding 1 × 10^12^ C_60_^+^/cm^2^ and decreased above 1 × 10^13^ C_60_^+^/cm^2^ due to the highly efficient sputtering. It should be noted that the elongation is induced even with 1 MeV C_60_ ions.

XTEM observations clearly showed not only the elongation of NPs but also a significant loss of the SiO_2_ layer. The significant loss was also confirmed by RBS and even the naked eye as loss of the NP layer’s color. Since *S*_e_ increased and *S*_n_ decreased in the energy region between 1 and 6 MeV, the observed sputtering that increased with increasing energy was ascribed to the electronic origin. However, the observed magnitude was higher by more than one order than the empirical cubic law^27^, *Y* = *B*_1_*S*_e_^3^ for electronic sputtering by monomer SHIs, indicating the cluster enhancement and/or the synergy effect^17,33,34^.

Ion tracks and possible hillocks caused by C_60_ ion irradiation were observed in crystalline SiO_2_ by XTEM observations. The tracks of 10.4 ± 3.0 nm in diameter and 60–80 nm in length were formed under 4 MeV C_60_ ion irradiation. The track length was much shorter than those associated with SHIs having the same *S*_e_. To understand whether the elongation of NPs was induced in a layer deeper than the ion tracks, a SiO_2_ layer of 200 nm was deposited on the NP layer and irradiated with 6 MeV C_60_ ions. The NPs were rarely elongated in this configuration where the NPs were not touched by any ion tracks but impacted with C_60_ ions at ~2.5 MeV if they could not be fragmented. This observation confirmed that most of the C_60_ ions were fragmented beyond the track length. Since the fragments were too small and too separated in the region deeper than the track lengths, cooperative excitation between the fragments were no longer expected.

## Methods

### Samples

Two types of NP samples were prepared: (i) Au NPs were formed in amorphous SiO_2_ by sequential vacuum depositions and annealing: At first, 3 nm thick of Au film was deposited on SiO_2_ substrate by electron beam vaporization. Rapid thermal annealing at 300 °C for 10 minutes transformed the continuous Au film to isolated NPs. Then the NPs were covered with SiO_2_ film of 100 nm thick by sputtering deposition. See supplementary materials. (ii) Zn NPs were formed by implantation of 60 keV Zn ions to SiO_2_ to a fluence of 1 × 10^17^ Zn^+^/cm^2^. Even without post thermal annealing, Zn NPs of 10.3 ± 2.3 nm in diameter were formed in the depth region between 20 and 70 nm.

### Irradiations

The irradiation of C_60_^+^ ions was carried out at Takasaki Advanced Radiation Research Institute (TARRI), National Institutes for Quantum and Radiological Science and Technology (QST), using a 3 MV tandem accelerator and a newly developed high-flux C_60_ negative ion source. While the samples were irradiated with C_60_ positive ion beams of four different energies, 1, 2, 4, and 6 MeV, the beam current was utilized at ~50 pA for 6 MeV and ~100 pA for other energies through an aperture of 3 mm in diameter. Some samples were irradiated with an incident angle of 45°, to detect the shape elongation of NPs by the optical linear dichroism (OLD) spectroscopy. Crystalline SiO_2_ samples were irradiated with 0 or 7° from the surface normal for ion-track observation by cross-sectional transmission electron microscopy (XTEM).

### Measurements

A standard dual-beam spectrophotometer was used for the OLD spectroscopy in the wavelength region of 215–800 nm with a resolution of 1 nm; a pair of optical polarizers (extinction ratio < 5 × 10^−5^ each) were used. A sample was set between the two polarizers, P and A, and illuminated by linearly polarized monochromatic light from the spectrophotometer through the first polarizer P. Light transmitted through the sample was detected through the second polarizer A, whose polarization plane was set to the same angle as that of polarizer P. The role of the second polarizer is to remove the birefringence signal. The optical transmittance was plotted in the form of optical density (OD = −log_10_ *T*) without correction of reflection, where *T* denotes the transmittance. An area of approximately 1 mm in diameter of the sample was illuminated through an aperture.

XTEM was carried out using JEM-2100 (for bright field observation) and JEM-2100F, JEOL (for scanning TEM and X-ray energy dispersive spectrometry mapping) under an acceleration voltage of 200 kV. The XTEM samples were fabricated with 30 keV Ga focused ion beam (FIB) milling. To identify the surface position in the cross-sectional configuration, thin layer of Pt was deposited on the sample surface before the FIB milling. Rutherford backscattering spectrometry (RBS) was carried out in TARRI to determine the content of Zn atoms in the sample using a 2 MeV He^+^ beam of 1 mm in diameter with a scattering angle of 165°. The data were analyzed with RUMP code.

## Supplementary information

Supplementary Information

## Data Availability

The datasets and materials generated during the current study are available from the corresponding author on reasonable request.
