# Supplementary material for: C60 ions of 1 MeV are slow but elongate nanoparticles like swift heavy ions of hundreds MeV
Source: Sci Rep. 2019 Oct 18;9:14980. doi: 10.1038/s41598-019-49645-5 (PMC6800440; doi:10.1038/s41598-019-49645-5)
Supplement: Supplementary file 1 — Supplementary Information [file 41598_2019_49645_MOESM1_ESM.pdf]

Supplementary Materials

C_{60} ions of 1 MeV are *slow* but elongate nanoparticles like *swift* heavy ions of hundreds MeV

H. Amekura [1)*], K. Narumi [2)], A. Chiba [2)], Y. Hirano [2)], K. Yamada [2)], D. Tsuya [1)],
S. Yamamoto [2)], N. Okubo [3)], N. Ishikawa [3)], and Y. Saitoh [2)]
[1)] *National Institute for Materials Science (NIMS), Tsukuba, Ibaraki, Japan*
[2)] *National Institutes for Quantum and Radiological Science and Technology (QST), Takasaki, Japan,*
[3)] *Japan Atomic Energy Agency (JAEA), Tokai, Ibaraki, Japan*

S1. Nanoparticle (NP) Preparation

1) Zn NPs by 60 keV Zn ion implantation

First, we planned to study the elongation of Zn NPs mainly using the optical dichroism, which were formed in amorphous SiO_2 by implantation of 60 keV Zn ions to 1×10^{17} ions/cm^2 [1]. As shown in Fig. 3(a) in the main text, the highest elongation is attained at 1E13 C_{60}/cm^2 from the optical data. Then we tried XTEM observations as shown in Fig. S-1. Comparing to the unirradiated sample, the NPs are aligned perpendicular to the surface, indicating very weak but the shape elongation we believed.

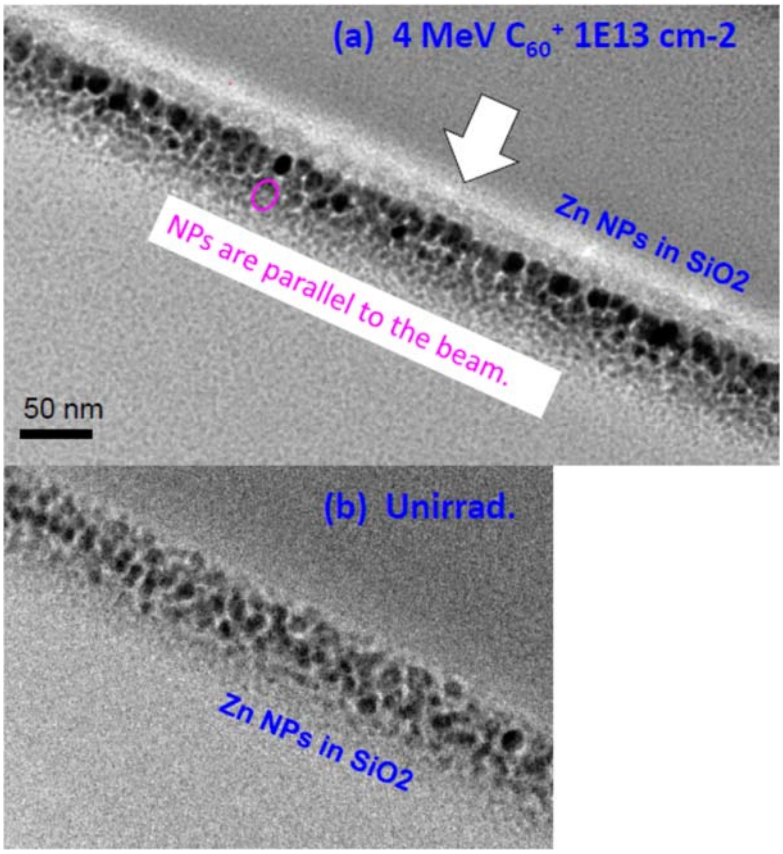

Fig. S-1. XTEM image of Zn NPs in SiO_2 irradiated with 4 MeV C_{60} ions to 1E13 C_{60}/cm^2 (upper), and that of unirradiated one (lower).

However, the majority of readers might suspect this

elongation. However, we cannot increase the fluence. The optical data suggest that further increase of the fluence turns to the destruction of elongated NPs.

2) Au NPs by sequential deposition and annealing

Then we decided to make another type of NPs, i.e., Au NPs by sequential deposition of Au and SiO_2 and annealing as shown in Fig. S-2. Since only the edge-on images are shown in the text, here the plain views of the NPs are shown. Figure S-2(a) shows a SEM image of the NPs before SiO_2 layer deposition. Isolated NPs are observed. Figure S-2(b) shows XTEM

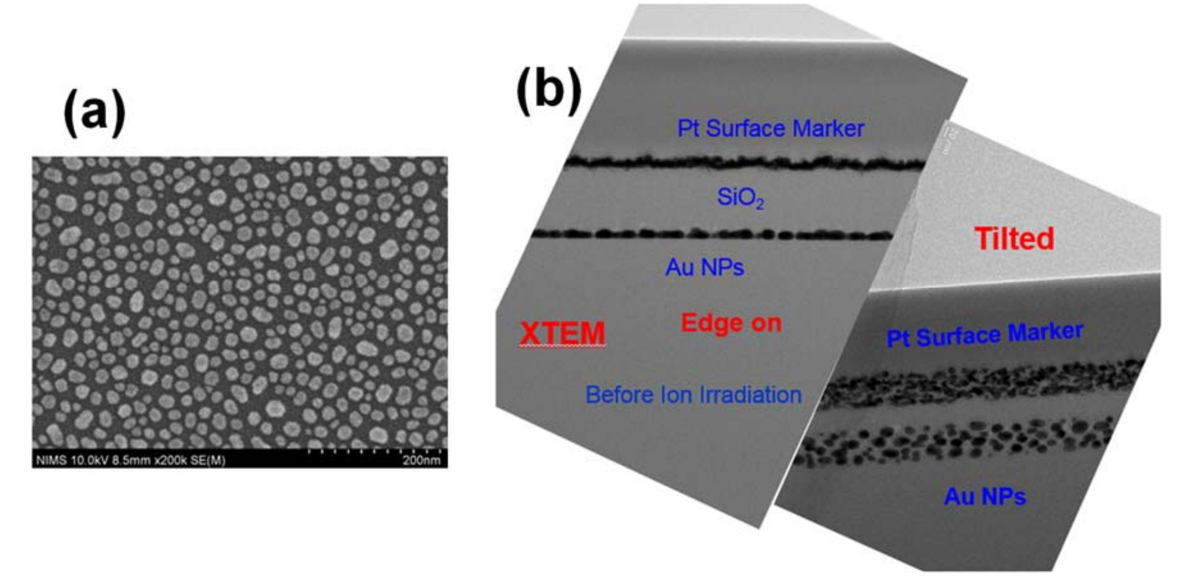

Fig. S-2. (a) SEM image of Au NPs formed by deposition of 3 nm thick Au film and rapid thermal annealing at 300°C for 10 minutes in Ar atmosphere. (b) After the formation of Au NPs, a SiO_2 layer of 100 nm thick was deposited. XTEM sample was fabricated by FIB milling. The XTEM images of the edge-on and tilted are shown.

images, edge-on (left) and tilted (right), from the same sample. Also the isolated NPs are confirmed.

According to Rizza et al. [2], the saturated aspect ratio $L/2R_o$ is proportional to

$$\frac{L}{2R_o} \propto R_o^2$$

which is proportional to the square of the initial diameter R_o, where L means the length of nano-rods. While the mean size of Zn NPs is ~10nm, that of the Au NPs is ~20nm. Consequently,

the Au NPs showed stronger elongation, while it is not sure whether the saturation is attained or not. However, the NPs are not spherical but disk-shaped. Since optical dichroism method is precisely applicable only to spheres and prolate spheroids, This kind of NPs have problems.

However, this kind of Au NPs has another problem. This is shown in Fig. S-3, which shows the absorption spectra at different fluences. Up to the fluence of 2.5E13 C_{60}/cm^2, the elongation is weak. At 5E13 C_{60}/cm^2, a strong elongation was detected by the optical method. This is the sample observed by TEM.

However, if we increases the fluence a little bit, i.e., to 7E13 C_{60}/cm^2 , not only the elongation, i.e., difference between the solid and dotted curves, but also the peak disappeared. All the Au NPs were sputtered out. Since all the NPs located at the same depth, all the Au NPs disappear simultaneously. Therefore the selection of the fluence is difficult. Contrary, Zn NPs distributed in wider depth region, which is better for the stable observation.

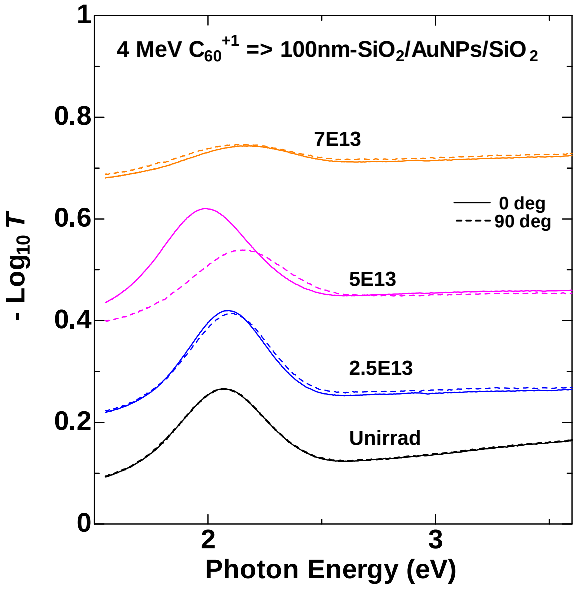

Fig. S-3. Optical spectra of Au NPs in SiO_2 for different polarization (solid curves and dotted curves).

Consequently, we have studied the elongation of Zn NPs using the optical method at the energies of 1, 2, 4, and 6 MeV. The TEM observation was carried out on the sample irradiated with 4 MeV only.

S2. Electron beam induced amorphization of c-SiO_2 under TEM observation

It should be noted that c-SiO_2 irradiated with C_{60} ions exhibited high susceptibility to amorphization even against the 200 keV electron beam irradiation for TEM observation. After long time exposure by high flux electron beam in TEM, a c-SiO_2 sample has exhibited amorphization and simultaneous loss of the track images. It seems that the crystallinity of the sample is a prerequisite for the observation of the tracks, since the tracks are observed as a disturbance of electron beam going through the periodic crystalline structure. This susceptibility makes the high resolution TEM observation of tracks difficult.

Quartz is known to form self-trapped excitons (STE) as similar to the amorphous SiO_2 and alkali halides. Some portion of STEs induce non-radiative recombination and form defects. This defect formation is not due to displacements by electrons. It is defect formation by purely electronic excitation. Itoh et al. [3] proposed that the formation of STEs is the origin of the very low track formation threshold of 2 keV/nm. Also the self-trapping is induced by assistant of defects (Extrinsic self-trapping) [4]. Since point defects were introduced during TEM observation, which may assist the extrinsic ST.

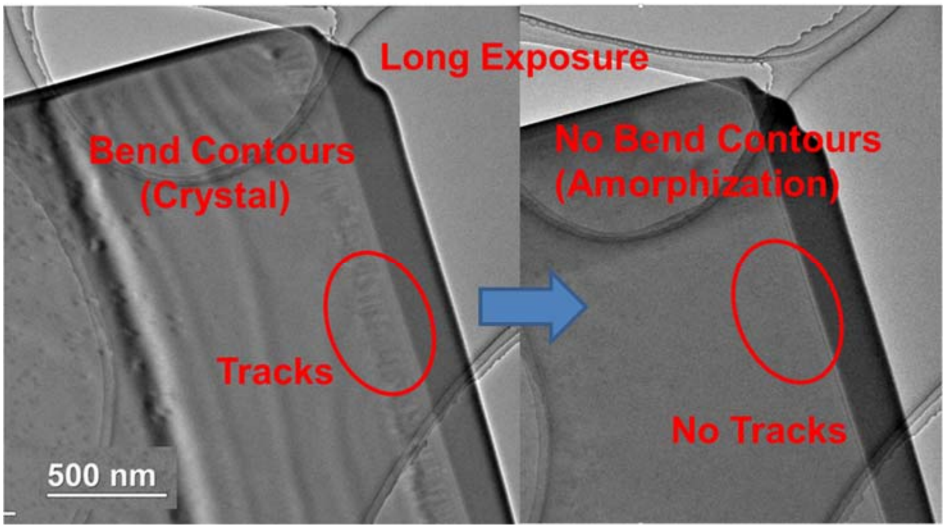

Fig. S-4. XTEM images of c-SiO_2 irradiated with 4 MeV C_{60} ions to 5×10^{11} C_{60}/cm^2. While ion tracks and bend contours were clearly observed in the beginning of TEM observation, they disappeared after long time exposure. Since the bend contours are indicator of the crystallinity, the long-time exposure induced the amorphization of the sample. The acceleration voltage for TEM observation was 200 kV.

[1] H. Amekura and N. Kishimoto, *Fabrication of oxide nanoparticles by ion implantation and thermal oxidation,* in *"Lecture Notes in Nanoscale Science and Technology" Vol. 5*, edited by Z. Wang (Springer, New York, 2009), p. 1~75.

[2] G. Rizza, P.E. Coulon, V. Khomenkov, C. Dufour, I. Monnet, M. Toulemonde, S. Perruchas, T. Gacoin, D. Mailly, X. Lafosse, C. Ulysse, and E.A. Dawi, *Rational description of the ion-beam shaping mechanism*, Phys. Rev. B **86,** 035450 (2012).

[3] N. Itoh, D.M. Duffy, S. Khakshouri, and A.M. Stoneham, *Making tracks: electronic excitation roles in forming swift heavy ion tracks*, Journal of Physics: Condensed Matter **21,** 474205 (2009).

[4] Y. Toyozawa, *Optical Processes in Solids* (Cambridge University Press, Cambridge 2003).
